# Curcumin Can Decrease Tissue Inflammation and the Severity of HSV-2 Infection in the Female Reproductive Mucosa

**DOI:** 10.3390/ijms21010337

**Published:** 2020-01-04

**Authors:** Danielle Vitali, Puja Bagri, Jocelyn M. Wessels, Meenakshi Arora, Raghu Ganugula, Ankit Parikh, Talveer Mandur, Allison Felker, Sanjay Garg, M.N.V. Ravi Kumar, Charu Kaushic

**Affiliations:** 1 Department of Pathology & Molecular Medicine and McMaster Immunology Research Centre, McMaster University, Hamilton, ON L8S 4K1, Canada; Danielle.Vitali@cihr-irsc.gc.ca (D.V.); bagrip@mcmaster.ca (P.B.); wessels@mcmaster.ca (J.M.W.); tsmandur@gmail.com (T.M.); felkea1@mcmaster.ca (A.F.); 2 Department of Pharmaceutical Sciences, College of Pharmacy, Texas A&M University, College Station, TX 77843, USA; arora@pharmacy.tamu.edu (M.A.); raghu@pharmacy.tamu.edu (R.G.);; 3 School of Pharmacy and Medical Sciences, University of South Australia, Adelaide 5000, Australia; ankit.parikh@unisa.edu.au (A.P.); Sanjay.Garg@unisa.edu.au (S.G.)

**Keywords:** curcumin, herpes, HSV-2, inflammation, pathology, nanoparticles, cytokine, CpG

## Abstract

Herpes Simplex Virus Type 2 (HSV-2) is one of the most prevalent sexually transmitted viruses and is a known risk factor for HIV acquisition in the Female Genital Tract (FGT). Previously, we found that curcumin can block HSV-2 infection and abrogate the production of inflammatory cytokines and chemokines by genital epithelial cells in vitro. In this study, we investigated whether curcumin, encapsulated in nanoparticles and delivered by various in vivo routes, could minimize inflammation and prevent or reduce HSV-2 infection in the FGT. Female mice were pre-treated with curcumin nanoparticles through oral, intraperitoneal and intravaginal routes, and then exposed intravaginally to the tissue inflammation stimulant CpG-oligodeoxynucleotide (ODN). Local intravaginal delivery of curcumin nanoparticles, but not intraperitoneal or oral delivery, reduced CpG-mediated inflammatory histopathology and decreased production of pro-inflammatory cytokines Interleukin (IL)-6, Tumor Necrosis Factor Alpha (TNF-α) and Monocyte Chemoattractant Protein-1 (MCP-1) in the FGT. However, curcumin nanoparticles did not demonstrate anti-viral activity nor reduce tissue pathology when administered prior to intravaginal HSV-2 infection. In an alternative approach, intravaginal pre-treatment with crude curcumin or solid dispersion formulations of curcumin demonstrated increased survival and delayed pathology following HSV-2 infection. Our results suggest that curcumin nanoparticle delivery in the vaginal tract could reduce local tissue inflammation. The anti-inflammatory properties of curcumin delivered to the vaginal tract could potentially reduce the severity of HSV-2 infection and decrease the risk of HIV acquisition in the FGT of women.

## 1. Introduction

Clinical and epidemiological evidence demonstrates that women are more susceptible than men to the acquisition of several sexually transmitted infections (STIs) [1,2]. In fact, heterosexual transmission in women is the fastest growing part of the Human Immunodeficiency Virus (HIV) pandemic (UNAIDS, 2014), with 40% of global infections occurring in the Female Genital Tract (FGT) [3]. Similarly, the global incidence of Herpes Simplex Virus 2 (HSV-2) infection is higher in women compared to men, with 14.8% of women being infected compared to 8% of men [4]. In addition to the social, economic and behavioral factors that contribute to the increased prevalence of HSV-2 and HIV in women, there is a biological basis for this increase in susceptibility.

Mucosal inflammation in the FGT is a biological mechanism that is associated with increased risk of HIV infection in women [5,6]. In particular, an inflammatory microenvironment is necessary for both the recruitment of HIV target cells (CD4^+^ T cells, macrophages, dendritic cells) to the FGT and the establishment of productive and systemic HIV infection [7]. To this point, we have shown that induction of pro-inflammatory cytokines by genital epithelial cells in response to bacterial and viral infections can result in the rapid impairment of mucosal barrier function in the genital epithelium, which may help HIV transverse the mucosal lining and infect underlying target cells [8,9,10]. Current research linking HSV-2 infection with increased susceptibility to HIV centers on the hypothesis that HSV-2 increases local production of pro-inflammatory cytokines and chemokines that facilitate HIV-target cell recruitment and contribute to mucosal barrier disruption [8,11,12]. Indeed, certain innate inflammatory responses are known to favor HSV-2 replication rather than restrict it. For example, activation of Nuclear Factor Kappa-B (NFκB), a transcription factor involved in the regulation of inflammatory gene expression during HSV infection, increases the efficiency of HSV-2 replication [13]. Taken together, these studies suggest that the targeting of inflammatory pathways in the FGT should be investigated as a possible prophylactic strategy for the prevention of both HIV and HSV-2 infection in women.

The compound curcumin (diferuloylmethane), which is the primary active constituent of turmeric, has been extensively studied for its anti-inflammatory activity in a variety of diseases in experimental and clinical studies [14]. Curcumin has been shown to exert anti-inflammatory actions by downregulating the activity of cyclooxygenase-2, lipoxygenase, and inducible nitric oxide synthase enzymes [15,16], inhibiting the production of pro-inflammatory cytokines (Tumor Necrosis Factor Alpha (TNF-α), Interleukin (IL)-1, -2, -6, -8 and -12, macrophage inflammatory protein (MIP), and monocyte chemoattractant protein-1 (MCP-1)) [17], and downregulating mitogen-activated and Janus kinases [18,19]. Anti-inflammatory activities are likely mediated by curcumin-mediated suppression of NFκB [16,17,20]. In our own studies, we examined whether curcumin treatment could reduce the production of cytokines and chemokines that induce an inflammatory microenvironment in the FGT capable of recruiting HIV target cells and impairing the mucosal barrier [21]. Results of these in vitro studies demonstrated that curcumin treatment abrogated the upregulation of the pro-inflammatory cytokines TNF-α and IL-6, and chemokines IL-8, RANTES and IL-10, and helped prevent barrier disruption following HIV exposure [21]. Curcumin also inhibited the indirect activation of the HIV long terminal repeat promoter by inflammatory factors in the presence of co-infecting STIs, and exhibited direct anti-viral activity against HSV-2, likely through the suppression of NFκB [21]. These findings suggest that modulating the microenvironment of the FGT with curcumin could possibly minimize genital inflammation and/or control against HSV-2 infection in vivo, which would assist in the prevention of HIV infection in the FGT.

Thus far, the therapeutic potential of curcumin has primarily been examined following systemic administration in vivo, but efficacy by this route of delivery is limited due to its rapid metabolism and conjugation in the liver [22,23,24]. To address bioavailability concerns, Poly(Lactic-Co-Glycolic Acid) (PLGA) polymeric nanoparticles are currently being used as oral delivery vehicles. Shaikh et al. showed that encapsulating curcumin in polymer nanoparticles improves peroral bioavailability by at least 9-fold compared to crude curcumin in rodents [25]. Furthermore, following oral and intraperitoneal (IP) delivery, nanocurcumin therapy can delay the progression of cataract development [26], protect against inflammatory markers and lipid metabolism in streptozotocin-induced diabetes, and have significant benefit in other disease models [27]. In addition, solid dispersion formulations of curcumin greatly increased its bioavailability and were shown to decrease cognitive impairment in an animal model of Alzheimer’s disease [28,29].

In the current study, our aim was to determine whether delivery of curcumin by various in vivo routes would reduce inflammation in the genital tract and prevent HSV-2 infection in vivo. Female C57BL/6 mice were pre-treated with curcumin nanoparticles either systemically (oral and IP delivery) or locally (intravaginal (IVAG) delivery) and exposed to immunostimulatory CpG-oligodeoxynucleotides (ODN). While no significant anti-inflammatory effects were observed following oral or IP delivery of the curcumin nanoparticles, we found that IVAG delivery of curcumin nanoparticles reduced both CpG-mediated inflammatory histopathology, as well as the production of pro-inflammatory cytokines in the genital tract. However, when mice were pre-treated with curcumin nanoparticles IVAG and then challenged with sub-lethal and lethal doses of HSV-2, curcumin did not demonstrate any direct anti-viral activity. In contrast, when mice were given solid dispersion formulations of curcumin IVAG, both reduced tissue pathology and increased survival were observed with HSV-2 infection. These findings demonstrate that curcumin nanoparticle delivery in the genital tract can mitigate inflammation in vivo, which indicates its potential use as a therapeutic to decrease the risk of HIV acquisition in women.

## 2. Results

### 2.1. Oral or IP Administration of Curcumin Nanoparticles Does Not Demonstrate Anti-Inflammatory Activity in the Genital Tract

We first tested the anti-inflammatory activity of curcumin nanoparticles in the FGT following oral or IP delivery. IVAG administration of CpG oligodeoxynucleotides (CpG-ODNs) was used as a model of acute inflammation in the mouse FGT. CpG-ODN have been shown to act as immunostimulants that potently activate the innate immune system through induction of the inflammatory transcription factor NFκB, and result in a thickened vaginal epithelium [30,31]. Significant immune cell infiltrate expressing CD11b^+^ and NK1.1^+^ was also reported following CpG-ODN administration [30], indicating infiltration of pro-inflammatory immune cells into the vaginal mucosa.

**Figure 1:**
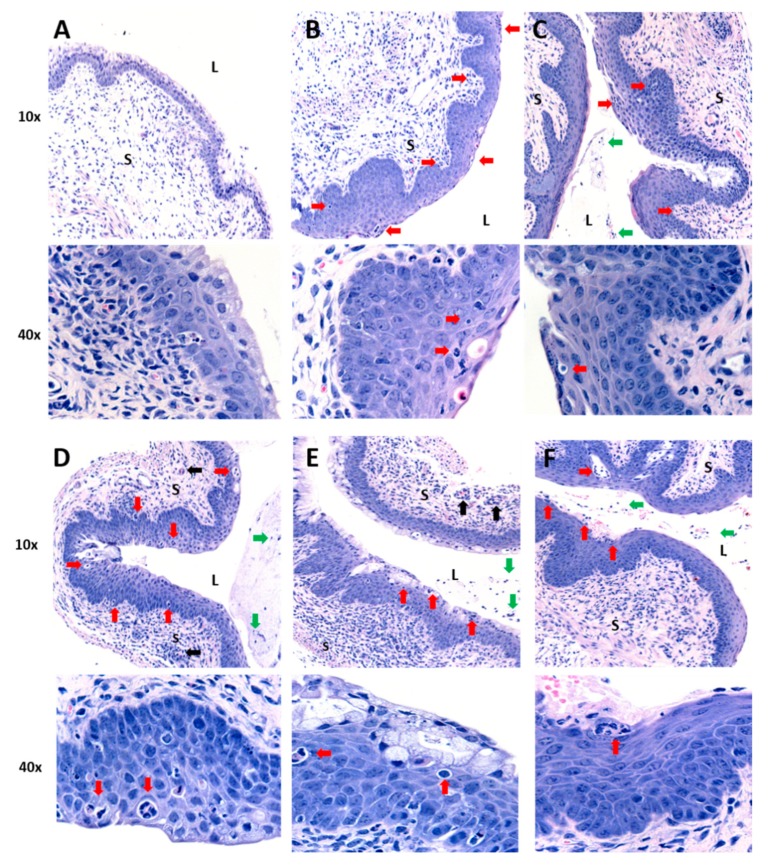
Histology of vaginal tissues from mice treated with curcumin nanoparticles by intraperitoneal or oral delivery and challenged with CpG-oligodeoxynucleotide (ODN). All mice were depo-treated (*n* = 3–6 per group). (**A**) Mice received water with no CpG-ODN (negative control). All other groups (**B**–**F**) received intravaginal (IVAG) inoculation with 30 µg CpG-ODN 4 h after primary treatment with: (**B**) water (positive control), (**C**) 1.25 mg of curcumin in nanoparticles IP, (**D**) vehicle-only nanoparticles IP, (**E**) 1.25 mg of curcumin in nanoparticles orally, or (**F**) vehicle-only nanoparticles orally. Vaginal tissues were fixed in formalin, sectioned and stained with H&E. Images are representative from vaginal tissue of 1 animal per treatment group, with magnifications of 10× and 40×. L denotes vaginal lumen and S denotes the vagina submucosa. Red arrows indicate thickened vaginal epithelium and/or inflammatory cell infiltration in the epithelium and submucosal tissue. Black arrows indicate inflammatory cell infiltrates in the blood vessels. Green arrows indicate inflammatory cells in the luminal space.

To assess and quantify the anti-inflammatory activity of curcumin nanoparticles delivered by systemic routes, female C57BL/6 mice received a single 1.25 mg dose of curcumin within PLGA nanoparticles, equivalent PLGA nanoparticles with no curcumin (vehicle-only control), or the same volume of water alone as a control, via oral gavage or IP injection. This was followed 4 h later by IVAG delivery of CpG-ODN (30 µg). Previous data indicate that blood levels of curcumin peak between 2 h and 4 h following oral delivery using nanoparticles [25]. Mice were sacrificed 24 h following CpG-ODN administration and vaginal tissue was collected and processed for hematoxylin and eosin (H&E) staining (Figure 1). Assessment of histopathology indicated that while vaginal tissue from mice without any CpG treatment had normal epithelial thickness and no cellular infiltrate (Figure 1A), positive control mice challenged with CpG-ODN had extensive thickening and immune cell infiltrates (Figure 1B). In addition, mice treated with curcumin nanoparticles had similar inflammatory pathology to the positive control (Figure 1C,E), as did tissues from mice given nanoparticles without curcumin (Figure 1D,F). The histopathology was visually similar regardless of oral versus IP delivery of nanoparticles.

Pathology scores on a scale of 0–3 were determined based on epithelial layer thickening, and infiltration of immune cells within the epithelium, the submucosa, blood vessels and lumen. There was no significant difference in average pathology score between the positive control (CpG-ODN only) (average score = 1.67) and mice that received curcumin nanoparticles via oral gavage (average score = 1.67) prior to CpG-ODN administration (Table 1). Although the average pathology score for tissues from mice receiving curcumin nanoparticles via IP route was lower (average score = 0.8) than the positive control with some mice showing no pathology, this was not statistically significant. In comparison, negative control mice that did not receive CpG-ODN showed near complete absence of tissue inflammation (average score = 0.33). Additional controls including vehicle-only nanoparticle preparations (no curcumin) tested via IP injection or oral gavage also had no significant anti-inflammatory activity (average scores 1.2 and 1.16, respectively). Considering that IP treatment with curcumin nanoparticles indicated some effect on pathology in some mice, we examined if higher doses (2.4 mg and 4.8 mg) of curcumin within nanoparticles would result in clear inhibition of inflammatory pathology. However, results showed no significant reduction in CpG-mediated inflammation (average scores = 1.16 and 1.5, respectively) compared to the positive control. Our results indicate that curcumin nanoparticles did not exert consistent and substantive anti-inflammatory effects in the vaginal tract when administered via IP or oral routes.

### 2.2. IVAG Delivery of Curcumin Nanoparticles Reduces Tissue Inflammation and Inflammatory Cytokines in the Genital Tract

**Figure 2:**
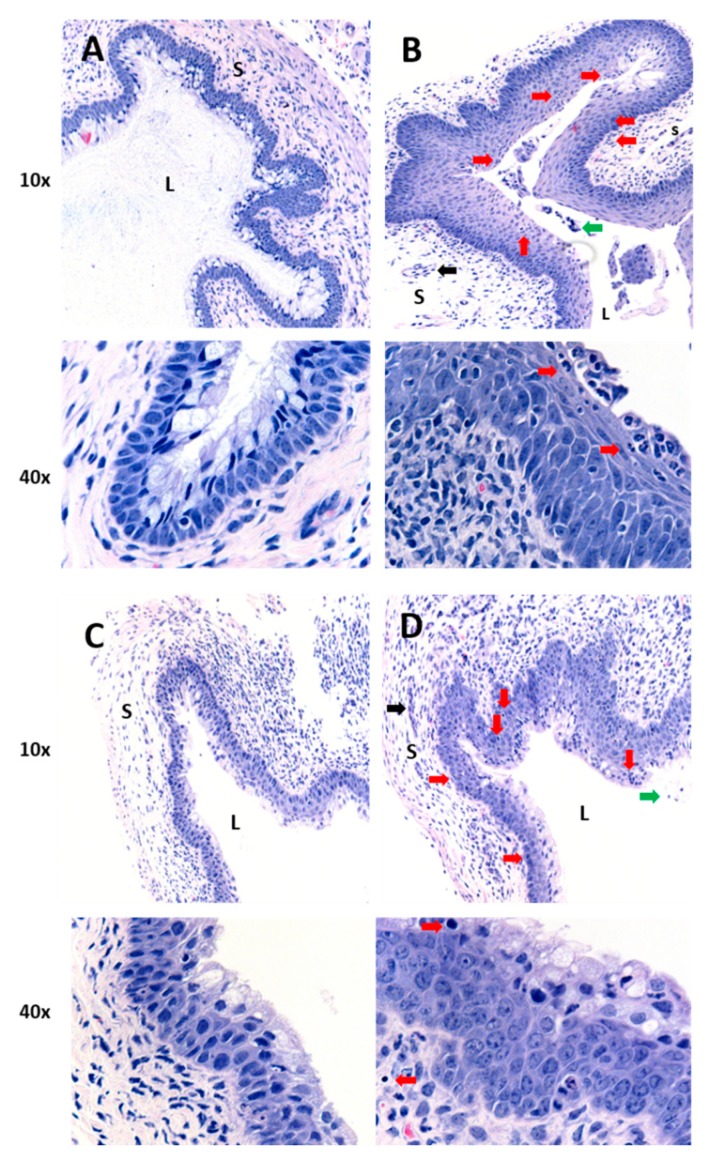
Histology of vaginal tissues from mice treated with curcumin nanoparticles by intravaginal delivery and challenged with CpG-ODN. All mice were depo-treated (*n* = 3–4 per group). (**A**) Mice received water with no CpG-ODN (negative control). All other groups (**B**–**D**) received IVAG inoculation with 30 µg CpG-ODN 2 h after primary treatment with: (**B**) water (positive control), (**C**) 0.5 mg of curcumin in nanoparticles IVAG, or (**D**) vehicle-only nanoparticles IVAG. Images are representative from vaginal tissue of 1 animal per treatment group, with magnifications of 10× and 40×. L denotes vaginal lumen and S denotes the vagina submucosa. Red arrows indicate thickened vaginal epithelium and/or inflammatory cell infiltration in the epithelium and submucosal tissue. Black arrows indicate inflammatory cell infiltrates in the blood vessels. Green arrows indicate inflammatory cells in the luminal space.

Because oral and IP routes of curcumin administration did not dampen CpG-mediated inflammation in the vaginal tract, IVAG administration was tested, since direct local administration in the FGT may provide more effective delivery. However, this route of administration restricts the dose compared to oral and IP because of the low volume that can be administered without resulting in leakage from the vaginal vault. Nevertheless, it would lead to higher target tissue bioavailability. Mice received either water, curcumin-loaded nanoparticles, or vehicle-only nanoparticles 2 h prior to IVAG inoculation with CpG-ODN. The negative control mice then were administered water instead of CpG-ODN, whereas the positive control mice received CpG-ODN. Vaginal tissues were removed 24 h after CpG-ODN treatment and inflammatory pathology was evaluated (Figure 2 and Table 2). Histologic images indicated that vaginal tissues from mice receiving IVAG treatment with curcumin nanoparticles had very few features of inflammation and were similar to tissues taken from negative control mice (Figure 2A,C), whereas inflammation was clearly evident in tissues from mice treated with vehicle-only nanoparticles (no curcumin) and then CpG-ODN or the positive control mice that received CpG-ODN alone (Figure 2B,D). Mice that were treated IVAG with the curcumin nanoparticles had statistically significant lower scores for tissue inflammation (average score = 0.5) compared to mice that received vehicle-only nanoparticles (average score = 1.67) or the positive control mice (average score = 2.7), and were not significantly different from negative control mice (Table 2).

**Figure 3:**
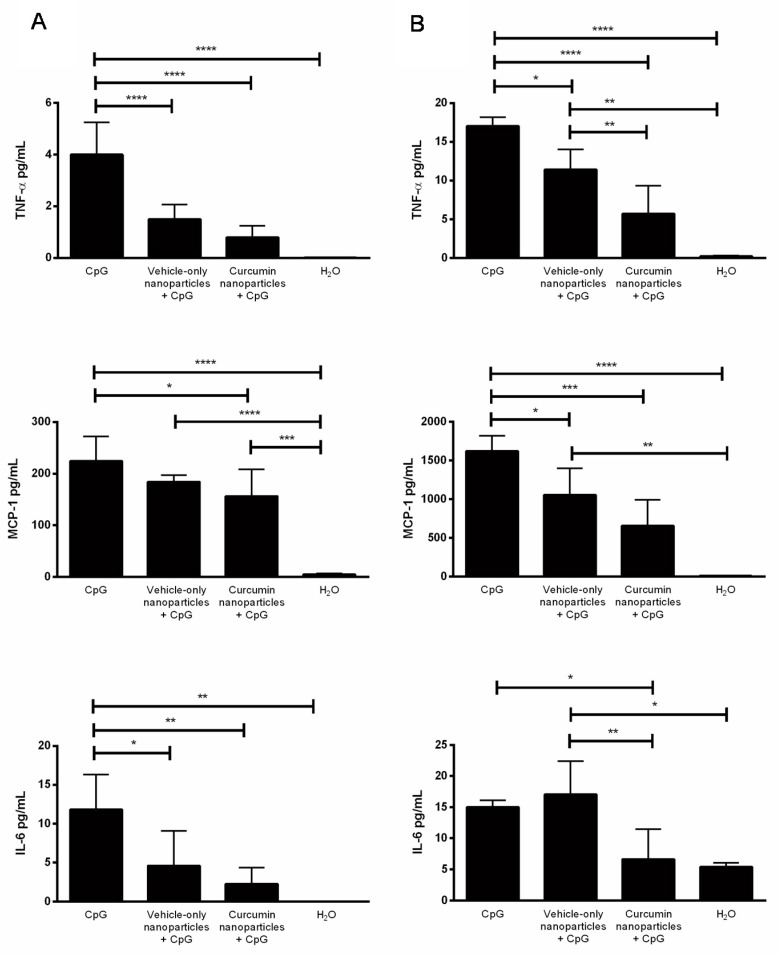
Anti-inflammatory effects of curcumin nanoparticles on inflammatory cytokine production following intravaginal delivery. Depo-treated mice (n = 3–4) received either water (H_2_O), nanoparticles without curcumin (vehicle-only), or nanoparticles with 0.5 mg of curcumin IVAG 2 h prior to inoculation with 30 μg of CpG-ODN. Negative control mice received water in the absence of CpG-ODN. (**A**) Cervical tissues and (**B**) vaginal tissues were excised and homogenized, and levels of Tumor Necrosis Factor Alpha (TNF-α), Monocyte Chemoattractant Protein-1 (MCP-1) and Interleukin (IL)-6 were measured in tissue homogenate supernatants. Data were analyzed by one-way ANOVA. * *p* < 0.05; ** *p* < 0.01; *** *p* < 0.001; **** *p* < 0.0001. Data are expressed as the mean + S.D. pg/mL.

Vaginal and cervical tissues from the same groups of mice were collected and homogenized for cytokine analysis. Both cervical and vaginal tissues from mice receiving curcumin delivered by nanoparticles IVAG showed significantly lower concentrations of pro-inflammatory cytokines TNF-α, MCP-1 and IL-6, compared to positive controls that received CpG-ODN alone (Figure 3). This demonstrated that IVAG delivery of curcumin nanoparticles, can facilitate significant reduction in inflammatory cytokines throughout the genital tissue in response to CpG-ODN. Interestingly, treatment with vehicle-only nanoparticles also appeared to decrease some of the cytokine levels in both tissues. However, curcumin nanoparticles significantly decreased levels of all cytokines, compared to CpG-ODN alone.

### 2.3. Nanoparticles Localize within the Vaginal Tissue Following IVAG Administration but Not after IP Delivery

**Figure 4:**
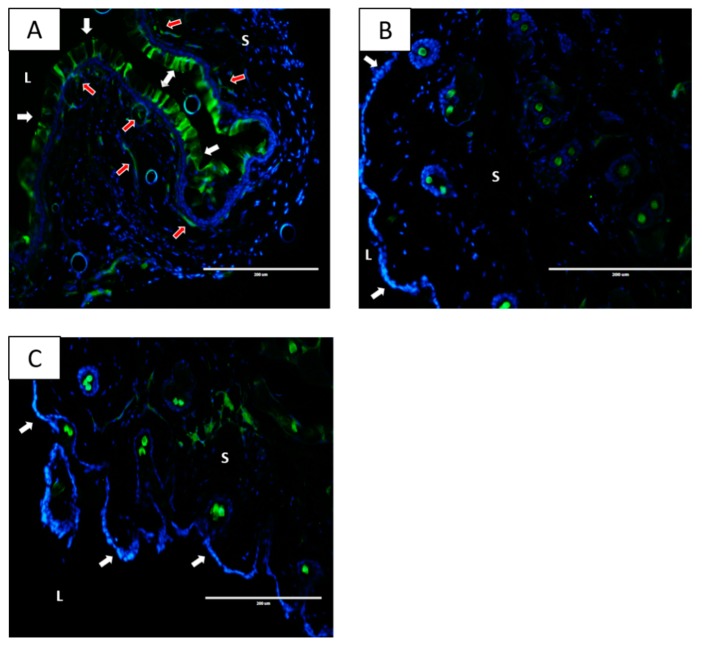
Vaginal tissue distribution of nanoparticle preparations following intraperitoneal and intravaginal delivery. (**A**) Mice received fluorescein-Poly(Lactic-Co-Glycolic Acid (PLGA) nanoparticles IVAG and vaginal tissue was excised 2 h later. (**B**) Mice received fluorescein-PLGA nanoparticles via IP injection and vaginal tissue was excised 24 h later. (**C**) H_2_O was administered IP as a negative control and vaginal tissue was excised 24 h later. Vaginal tissues were sectioned and observed under an EVOS fluorescent microscope with a green filter. L denotes vaginal lumen and S denotes the vagina submucosa. White arrows indicate the vaginal epithelial lining. Red arrows indicate subepithelial penetration of the nanoparticles into the submucosal tissue. Representative images from a single experiment with three animals per condition are shown (Magnification 20×). Fluorescent spots seen within tissues are likely auto-fluorescent neutrophils.

The ability of IVAG-delivered curcumin nanoparticles to reduce CpG-ODN-induced inflammation may be due to enhanced localization of those nanoparticles to the epithelial layers of the FGT. Considering that IP delivery provided no significant anti-inflammatory effect, we examined the tissue distribution pattern of nanoparticles following IP or IVAG delivery. To image the tissue distribution of the nanoparticles, we used fluorescein-labeled PLGA nanoparticles, because the curcumin nanoparticles were constructed using PLGA. Mice received fluorescein-labeled nanoparticle or water by IVAG delivery, and vaginal tissues were collected (Figure 4). Other groups received fluorescein-labeled nanoparticle, or water, through IP delivery, and vaginal tissues were collected. Tissues were processed and examined by fluorescence microscopy. The nanoparticles were well absorbed and retained within the vaginal epithelium 2 h following IVAG delivery (Figure 4A). There is also evidence of subepithelial penetration of the nanoparticles following IVAG delivery, indicated by fluorescent bands below the vaginal epithelial lining in the submucosal tissue. Conversely, there was no evidence of nanoparticles in the vaginal tissue up to 24 h following IP injection (Figure 4B). Thus, localization of nanoparticles to the vaginal epithelium correlates with the ability of the curcumin nanoparticles to reduce inflammation when delivered IVAG.

### 2.4. Curcumin Nanoparticles Do Not Confer Protection against High- or Low-Dose IVAG Challenge with *HSV-2*

**Figure 5:**
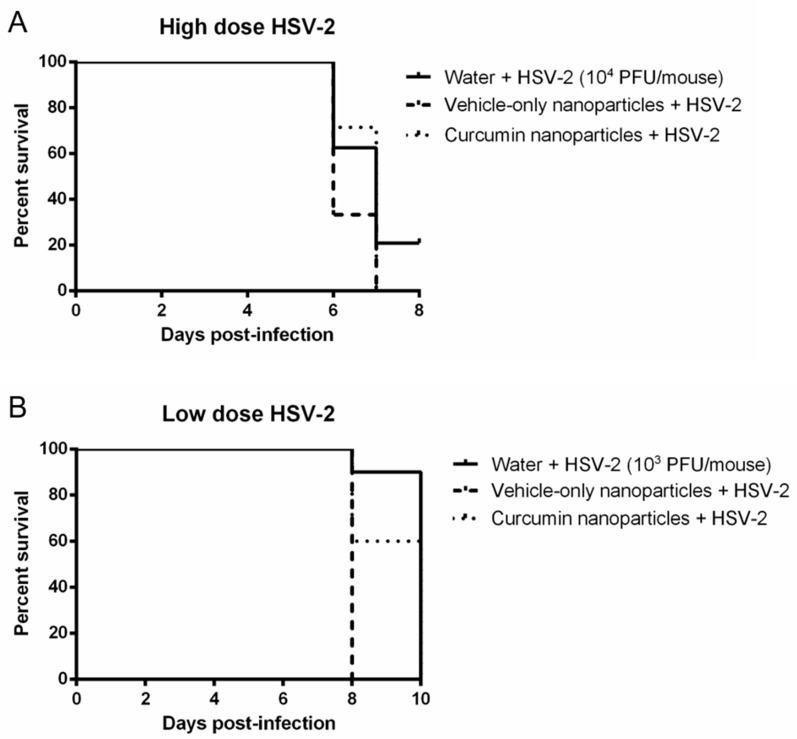
Survival of mice in response to high- and low-dose Herpes Simplex Virus Type 2 (HSV-2) infection when treated with curcumin nanoparticles. Depo-treated mice (*n* = 5–10 per group) received either water, 0.5 mg vehicle-only nanoparticles or 0.5 mg curcumin in nanoparticles IVAG, 2 h prior to inoculation with HSV-2 at (**A**) high dose (10^4^ PFU/mouse) or (**B**) low dose (10^3^ PFU/mouse). Survival was monitored for 8–10 days post-infection and is displayed as % survival per day post-infection.

Previous in vitro studies revealed that curcumin treatment significantly decreases HSV-2 replication in cultures of primary genital epithelial cells through the inhibition of NFκB [21]. Since we observed in vivo anti-inflammatory effects following IVAG delivery of curcumin in the CpG-ODN treated mice, we next tested whether this treatment would result in protection against a lethal, high-dose HSV-2 infection. Mice received IVAG a single 0.5 mg dose of curcumin in nanoparticles or vehicle-only nanoparticles 2 h prior to inoculation with a lethal dose (10^4^ PFU/mouse) of wild type (WT) HSV-2, and survival and genital pathology were monitored post-infection (Figure 5A). There was no clear difference in survival rates among the three treatment groups of mice, and all treatment groups showed similar levels of viral shedding (10^5^–10^6^ PFU/mouse) within 2 days of infection (data not shown).

All mice treated with curcumin nanoparticles showed extensive disease pathology over 8 days post-infection (Table 3). This degree of pathology and the onset time (1–3 days) was not different from mice given vehicle-only nanoparticles, nor the untreated, HSV-2 infected, positive control mice. These data indicated clearly that the IVAG treatment with curcumin nanoparticles offered no direct anti-viral protection against high infectious dosage of HSV-2.

We then conducted a similar experiment using a low dose of virus (10^3^ PFU/mouse) to determine if the curcumin nanoparticles might provide some measure of protection such as increased survival or reduced disease pathology during lower exposure to virus. No differences in survival (Figure 5B) or pathology (Table 3) were observed between vehicle-only and curcumin-treated mice despite the lower viral dose. Although there was some delay in the onset of pathology and cumulative pathology scores between the high and low viral dose groups (4–6 days for low dose compared to 1–3 days for high dose), this would be expected with lower dosage infection. Combined, these results indicate that the anti-inflammatory effects of curcumin delivered in nanoparticles do not translate into any substantive anti-viral activity against HSV-2 in vivo, regardless of the challenge dose of virus.

### 2.5. IVAG Pretreatment with Crude and Solubilized Curcumin Increases Survival Time for HSV-2 Infection and Reduces Pathology

**Figure 6:**
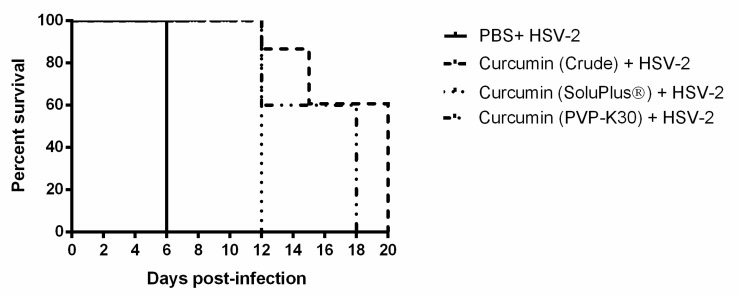
Survival of mice in response to high-dose HSV-2 infection after treatment with crude or solubilized curcumin. Depo-treated mice (n = 5–15 per group) received IVAG inoculation with either PBS or 1 mg of crude curcumin or 100 µg of curcumin in PVP-K30- or Soluplus^®^-based solid dispersion formulations and, after 6 h, were infected with 5 × 10^4^ PFU/mouse of HSV-2 virus IVAG. Survival was monitored for 20 days post-infection and is displayed as % survival per day post-infection. Differences were statistically significant by the Mantel–Cox log-rank test: HSV-2 alone vs. crude curcumin: *p* < 0.0001, HSV-2 alone vs. Soluplus^®^ curcumin: *p* = 0.0027, HSV-2 alone vs. PVP-K30: *p* = 0.0002, PVP vs. Soluplus^®^: *p* = 0.0308, Crude vs. Soluplus^®^: *p* = 0.0006, and Crude vs. PVP: *p* = 0.0457.

The absence of any substantive increase in survival or reduced pathology in response to HSV-2, after IVAG pre-treatment of mice using curcumin nanoparticles, may indicate an insufficient or a quickly diminished anti-inflammatory effect by curcumin delivered in this manner. For comparison, we decided to test crude extracts of curcumin. This approach is complicated by the fact that crude curcumin contains a large fraction of insoluble curcumin that may not be accessible to the FGT epithelial tissues. We therefore also utilized 2 known polymers, PVP-K30 and Soluplus^®^, to increase the solubility of crude curcumin by preparing solid dispersion formulations [29]. Groups of 4–6 mice received IVAG either PBS or 1 mg of crude curcumin in PBS or 100 µg curcumin in either solvent, 6 h before infection with a lethal dose of HSV-2. Survival and disease pathology were then monitored for 20 and 12 days, respectively. As can be seen in Figure 6, survival time was increased significantly for all groups of mice treated IVAG with high-dose curcumin by any formulation. While untreated, infected mice died by 6 days after treatment, the curcumin-treated mice did not succumb to HSV-2 until 12 days or later. When comparing the different formulations, mice treated with crude curcumin and PVP-K30 formulation had longest survival (20 and 18 days, respectively). However, the cumulative pathology scores were lowest in Soluplus^®^ followed by PVP formulation (average pathology score per mouse was 11.8 and 12.0, respectively). Cumulative pathology scores also indicated delayed onset of pathology and reduced average pathology score for all three groups of curcumin-treated mice, although the mice administered solid dispersion formulations exhibited the longest delay for onset of pathology and the lowest average scores (Table 4). Based on survival and pathology combined, PVP-K30-treated mice showed the best outcome, longest survival and lowest pathology. Thus, curcumin delivered IVAG in an enhanced soluble state does delay infection, pathology and death for mice infected with HSV-2.

## 3. Discussion

In this study, we describe for the first time a system of in vivo curcumin delivery that offers protection against inflammation in the FGT. Our results show that delivery of curcumin nanoparticles directly into the vaginal tract abrogated CpG-induced inflammatory vaginal pathology and diminished the production of pro-inflammatory cytokines IL-6, TNF-α and MCP-1 in vaginal and cervical tissues. This effect was not observed when curcumin nanoparticles were delivered by systemic routes of delivery, including oral gavage and IP injection, likely due to limited accumulation of the nanoparticles within the vaginal tract. Despite potent anti-inflammatory activity, prophylactic IVAG administration of curcumin nanoparticles did not reduce HSV-2 acquisition or genital pathology, when compared with vehicle-only, and untreated control groups. This lack of efficacy was consistent across both lethal (10^4^ PFU/mouse) and sub-lethal (10^3^ PFU/mouse) viral doses in our mouse model, suggesting that previously observed in vitro anti-viral properties [21] do not translate in vivo when curcumin is delivered by nanoparticles. In contrast, IVAG delivery of relatively large dosages of crude and solid dispersion formulations of curcumin resulted in delayed and/or reduced pathology as well as increased survival of mice challenged with HSV-2. The reason for the lack of effectiveness of curcumin nanoparticles against HSV-2 infection, despite evidence that curcumin nanoparticles can reduce inflammatory cytokines in the FGT, is unclear. It might relate to whether the amount of curcumin loaded in nanoparticles reached an effective dosage, an issue in the use of nanoparticles that needs to be improved. That may explain why the crude and solid dispersion formulations were more effective. More focus on formulations that increase the bioavailability of curcumin seem necessary to study their effects on inflammation in future research. Furthermore, our observations highlight the continuing need to test novel anti-viral intervention strategies that show promise in in vitro cell culture studies using more physiologically relevant and complex in vivo models.

Although the HSV-2 infection model in mice is well developed and useful in regard to HSV-2 infection, disease prevention in mice is not as dependent on the inflammatory state of the genital tissue prior to infection, whereas mucosal inflammation is understood to be a factor that contributes to HIV infection. Mucosal inflammation enhances the rate of sexual transmission of HIV in the FGT [5,6,32,33]. Recently, Masson et al. observed a three-fold increased risk of HIV infection in South African women with elevated levels of at least five mucosal pro-inflammatory cytokines [5]. Furthermore, Arnold et al. found increased frequencies of key HIV target cells, CD4^+^ T cells, in the endocervix of women with pro-inflammatory cytokine profiles [33]. Indeed, an innate and adaptive inflammatory cascade following viral exposure in the FGT is necessary for the recruitment of target cells to the site of exposure and the establishment of a productive, systemic infection [34]. Our lab has previously shed light on the mechanisms by which inflammation can facilitate viral transmission. Exposure to HIV envelope protein gp120 in vitro resulted in impairment of barrier function and significantly increased rates of viral transmission across the genital epithelium [9,10]. Given its potent anti-inflammatory properties, we previously examined if curcumin could be used in vitro to abrogate inflammatory processes that facilitate HIV acquisition in the FGT. Results of these in vitro studies demonstrated that curcumin treatment diminished the upregulation of pro-inflammatory cytokines and chemokines, prevented barrier disruption following HIV exposure and significantly decreased HIV replication in chronically infected T cells [21]. However, the efficacy and practical application of using curcumin to control inflammation in the genital tract in vivo were unclear.

This study is one of a few to investigate the efficacy of topical application of curcumin in the vaginal tract [35,36]. Although curcumin offered impressive benefits in in vitro preclinical studies, the translation into in vivo conditions has been difficult and the doses tested are often unfeasible to administer clinically [37]. Lack of clinical success with curcumin treatments is often linked to (a) poor solubility and (b) extensive intestinal and hepatic metabolic biotransformation resulting in poor oral bioavailability [37]. Thus, the focus of investigators has been to improve the therapeutic efficacy of curcumin in vivo by addressing both factors [37]. Polymeric nanoparticles have been actively explored as vehicles for oral delivery of pharmaceutically challenging compounds. Shaikh et al. have shown that encapsulating curcumin in polymer nanoparticles improved peroral bioavailability of curcumin by at least 9-fold compared to that of crude curcumin in rodents [25]. They have since published the ability of nanocurcumin to delay the progression of diabetic cataracts in rats with significantly higher therapeutic efficacy than crude curcumin [26]. The solid dispersion formulations also seem to enhance the solubility of curcumin. The Soluplus^®^ formulation used in other studies increased bioavailability by 117-fold and decreased cognitive impairment in a rat model of Alzheimer’s disease [28,29]. Future studies need to examine the underlying mechanism by which higher solubility formulations lead to decreased pathology, by examining both histopathology of the vaginal tract and presence of inflammatory cytokines, following HSV-2 infection after soluble curcumin treatment.

In more recent years, the therapeutic focus of curcumin nano-formulations has been diseases with an inflammatory link. Wang et al. showed that curcumin-solid lipid nanoparticles, administered by IP injection, effectively suppressed airway hyperresponsiveness, inflammatory cell infiltration and expression of IL-4 and IL-13 in bronchoalveolar lavage fluid in an asthma animal model [38]. The route of administration in the majority of other studies investigating similar therapeutic potentials is systemic, either by oral or IP delivery [39,40,41,42,43,44]. Our study is one of a few to investigate local administration of nanoparticles using topical application of curcumin in the genital tissue. We conclude from our study that this method of delivery results in significant anti-inflammatory benefits to the target area, which were not observed following systemic delivery. Our results using fluorescein tagged PLGA nanoparticles suggest that the curcumin nanoparticles can accumulate in the local epithelial tissues when given IVAG but are unable to accumulate in those tissues when given by oral or IP delivery. This likely explains the poor anti-inflammatory efficacy observed in vaginal tissue after IP or oral delivery.

The use of crude curcumin as a therapeutic agent in patients with HIV has been investigated in the past. A clinical trial in 40 patients over an eight-week period showed no significant reduction in patients viral load or elevation of CD4^+^ T cell counts, following oral administration of curcumin [45]. However, based on our findings we suspect that a solid dispersion formulation containing curcumin or curcumin nanoparticles could circumvent barriers in bioavailability and allow for retention of curcumin within the genital tract. It is our hope that this formulation will offer more desirable therapeutic outcome in clinical trials.

Additionally, there is renewed interest in the use of intravaginal rings to deliver pharmaceutical products into the genital tract [46]. These rings are designed to deliver sustained drug doses for extended periods of time while bypassing first pass metabolism in the gut [46]. Such rings are already established as an effective system to deliver hormonal contraceptives, but could be combined with microbicidal compounds like curcumin as a multipurpose prevention technology, offering protection against both unintended pregnancy and STIs like HIV [46]. However, as with any IVAG formulation, appropriate tissue concentrations of pharmacodynamically active drugs will have to be delivered in order to avoid any unintended alterations in the mucosal microenvironment or resident vaginal microbiota. These are all factors that should be investigated in a clinical setting before the implementation of such curcumin-based IVAG treatments into clinical trials for the prevention of HIV transmission in women.

In conclusion, our results indicate the promising potential of a topical curcumin formulation as a potent anti-inflammatory in the vaginal tract. Genital inflammation can increase HIV risk in women and contribute to the sequelae of chronic HIV infection. Thus, the curcumin nanoparticle formulation or a solubilized curcumin formulation could work in tandem with current prophylactic or anti-retroviral treatment strategies to have a significant impact on HIV infection and disease progression.

## 4. Materials and Methods

### 4.1. Preparation of Curcumin Nanoparticles 

Curcumin nanoparticles were prepared by a single emulsification process as described previously [25,26]. The process involved two steps, emulsification and evaporation. In brief, (a) the organic phase consisted of 500 mg of polymer (polylactide-co-glycolide-PLGA [50:50]) and 75 mg of curcumin in 30 mL ethyl acetate; (b) the aqueous phase consisted of 600 mg of polyvinyl alcohol (PVA) in 60 mL of water. The organic phase was emulsified into the aqueous phase followed by homogenization at 16,000 rpm for 30 min. This emulsion was then added into 200 mL of water to facilitate diffusion of organic solvent into aqueous phase that was subsequently evaporated over 3–4 h. The resultant suspension was then centrifuged at 15,000× *g* for 30 min at 4 °C. The resultant pellet was suspended in 25 mL of 5% *w/v* sucrose solution and frozen overnight at −80 °C. The freeze drying was carried out using bench top freeze drier (Labconco^®^ FreeZone^®^ Triad^®^ −85 °C Benchtop Freeze Dryers) at −55 °C for 54 h, followed heating at 20 °C for 20 h under vacuum (0.008 mbar). The freeze product was crimp sealed and stored at 4 °C until further use. The resulting PLGA nanoparticle preparations contained 0.15 mg curcumin per 1 mg of polymer and were in the size range of 290–300 nm. Dosages of curcumin nanoparticles given are described as mg of curcumin per dose.

For in vivo study of solid dispersion formulations, curcumin was obtained as a crude preparation with partial solubility (approx. 80% purity) from Sigma Aldrich (Sigma Aldrich, St. Louis, MO, USA). The crude preparations were solubilized using either of two amorphous polymer matrix/carriers, which are used widely to improve the solubility, dissolution rates and bioavailability of poorly soluble drugs. The first formulation, curcumin with Soluplus^®^, used the carrier polyvinyl caprolactam-polyvinyl acetate-polyethylene glycol graft copolymer, a matrix polymer that possesses both hydrophilic and lipophilic properties and has a high viscosity when solubilized [47]. The second formulation, curcumin with PVP-K30, used the carrier polyvinylpyroline K30, a hydrophilic matrix polymer that has a low viscosity when solubilized [48]. Given their increased solubility, we estimated that 100 μg/mouse of either solid dispersion formulation would exceed or match the biologically availability of the highest curcumin dosages (1.25 mg) in the nanoparticle preparations used.

### 4.2. Mice

The 6–8 week old female C57BL/6 mice were obtained from Charles River laboratories (Constant, QC, Canada). All mice were housed and maintained under specific pathogen-free and standard temperature-controlled conditions in the Central Animal Facility at McMaster University that followed a 12 h light/dark cycle. Mice were provided with low-fat mouse chow and water ad libitum. All animal studies performed were approved by and were in compliance with the Animal Research Ethics Board (AREB) at McMaster University and the Canadian Council on Animal Care (CCAC).

### 4.3. Depo-Provera Injection and Staging

Mice were subcutaneously injected with 2 mg of Depo-Provera (medroxyprogesterone acetate) as per previously published protocols [49]. Vaginal washes were collected daily for 4 consecutive days following Depo injection by pipetting 30 μL of PBS into and out of the vagina 5–6 times. Sample fluid was smeared on glass slides and examined by light microscopy to determine the stage of the estrous cycle, as previously described [49]. The following classification was used for identifying the stage of the cycle: estrus, >90% cornified epithelial cells; diestrus, >75% polymorphonuclear cells; meta-estrus, 50% epithelial cells, 50% polymorphonuclear cells. Mice treated with Depo remained in diestrus for 5 to 6 weeks.

### 4.4. Histomorphology of the Vaginal Tract Following CpG-ODN Treatment

To study the anti-inflammatory effect of curcumin in the vaginal tract, depo-treated mice were anesthetized and received curcumin nanoparticles at various time points prior to IVAG delivery of 25 μg of CpG-ODN or water as a control. The CpG-ODN used in these studies was ODN 1826, which is 20 nucleotides in length and contains two CpG motifs (5′-TCCATGACGTTCCT-GACGTT-3′) and has previously been delivered IVAG for immunostimulatory purposes [30,31]. After 24 h, vaginal tissue was removed, fixed in 4% paraformaldehyde, embedded in paraffin and sectioned at 7 µm for hematoxylin-and-eosin staining. Histopathology in the vaginal tract was scored for inflammation on the basis of the following four parameters: (1) thickness of the epithelium and extent of inflammatory cell infiltrate within the epithelium, (2) inflammatory infiltration in the submucosal tissue, (3) thickness and inflammatory infiltration in the blood vessels, and (4) inflammatory infiltration in the luminal space. A four-point scale was used: 0 indicated the absence of inflammatory pathology, evidenced by normal epithelium and minimal inflammatory cell infiltrate; 1 indicated some thickening of epithelium with occasional cell infiltrate to sub-epithelium; 2 indicated clear thickening of epithelium with infiltrate of sub-epithelium and some blood vessels; and 3 indicated severe inflammatory pathology, evidenced by substantive epithelial thickening and extensive inflammatory cell infiltrate in blood vessels and lumen. Average pathology for each experimental group was determined by adding the scores of each tissue and dividing by number of animals.

### 4.5. Multiplex Cytokine Assay

Murine vaginal tissue was collected and mechanically homogenized in PBS, using metal beads and the Gold BulletBlender system (Next Advance, Averill Park, New York, NY, USA). Homogenates were centrifuged at 8000 RPM for 5 min, and the supernatants were collected and stored at −80 °C until required. Cytokines and chemokines were quantified in duplicate in vaginal supernatants using the multiplex magnetic Luminex^®^ bead-based immunoassay using MagPix technology and the Mouse Cytokine Magnetic Kit 96-Well Plate Assay (Millipore, Billerica, MA, USA), as per the manufacturer’s instructions. The following cytokines and chemokines were measured: TNF-α, IL-6 and MCP-1. The range of detection of this assay was between 3.2 and 10,000 pg/mL.

### 4.6. HSV-2 Primary Inoculation

Depo-treated mice were anaesthetized via IP injection (150 mg of Ketamine/kg with 10 mg of Xylazine/kg) at a dose of 0.1 mL/10 g of body weight. Anaesthetized mice were gently swabbed intravaginally with sterile, dry cotton wool. Mice were then treated with either water or 0.5 mg of curcumin within nanoparticles delivered IVAG (kindly provided by Dr. Kumar, Texas A&M University). Two hours later, mice were anaesthetized as before and infected IVAG with 10 μL of a lethal (10^4^ PFU/mouse) or sub-lethal (10^3^ PFU/mouse) dose of WT HSV-2 strain 333. After inoculation, mice were placed on their backs for approximately 30–45 min to allow for the virus to infect the vaginal tract.

### 4.7. Genital Pathology Assessment during HSV-2 Infection

Genital pathology was monitored daily following HSV-2 infection and scored on a 5-point scale: 0, no infection; 1, slight redness of external vagina; 2, swelling and redness of external vagina; 3, severe swelling and redness of both vagina and surrounding tissue and hair loss in genital area; 4, genital ulceration with severe redness; 5, severe genital ulceration extending to surrounding tissue. Animals were considered to have reached end point when they reached pathology score of 4 and euthanized prior to reaching a score of 5. In order to compare pathology across the different groups, cumulative scores of pathology were determined by tabulating the number of mice with the highest score of pathology they achieved and the number of days that score was observed, as done previously [50]. Mice that did not survive the challenge were given the highest pathology score at the time of death. For the purpose of cumulative score calculation, the last day of the pathology scoring was determined when the first animal in a group reached a score of 5 or died. The sum of all scores for all the mice in a group was the total level of pathology for each group and then the average pathology score per mouse for each group was calculated.

### 4.8. Collection of Vaginal Washes

Vaginal washes were collected daily for 10 consecutive days following HSV-2 infection by pipetting 30 μL of PBS twice consecutively into and out of the vagina 5–6 times to give a total volume of approximately 60 μL, which was stored at −80 °C until required for testing.

### 4.9. Statistical Analysis

Statistical analysis and graphical representation were performed using GraphPad Prism 6.0d (GraphPad Software, San Diego, CA, USA). The Mantel–Cox log-rank test was used to calculate significant differences in survival. One-way analysis of variance (ANOVA) and Tukey’s multiple comparisons test was used to calculate significant differences in cytokine levels. The Kruskal–Wallis test of one-way ANOVA and Dunn’s multiple comparisons test was used to calculate significant differences in genital and inflammatory pathology scores.

## Figures and Tables

**Table 1:** Scoring of tissue inflammation following oral and intraperitoneal delivery of curcumin nanoparticles prior to inoculation with CpG-ODN.

Pre-Treatment	Dose ^d^	CpG-ODN	Mouse	Pathology Score	Average Score per Mouse (±S.D.)
Water (Oral)	0	N	1	0	0.33 (0.58) **^,^^
			2	1	
			3	0	
Water (Oral)	0	Y	1	2	1.67 (0.58) *
			2	1	
			3	2	
Vehicle-Only Nanoparticles (Oral)	0	Y	1	1	1.17 (0.52) *
			2	2	
			3	2	
			4	1	
			5	1	
			6	0	
Vehicle-Only Nanoparticles (IP)	0	Y	1	1	1.20 (0.84) *
			2	2	
			3	1	
			4	2	
			5	0	
Curcumin Nanoparticles (Oral)	1.25	Y	1	1	1.67 (0.52) *
			2	1	
			3	2	
			4	2	
			5	2	
			6	2	
Curcumin Nanoparticles (IP)	1.25	Y	1	0	0.80 (1.1) *
			2	2	
			3	0	
			4	2	
			5	0	
Curcumin Nanoparticles (IP)	2.4	Y	1	2	1.16 (0.75) *
			2	1	
			3	2	
			4	1	
			5	1	
			6	0	
Curcumin Nanoparticles (IP)	4.8	Y	1	0.5	1.50 (0.87) *
			2	2	
			3	2	

^d^—dose (mg) of curcumin in nanoparticles. Statistical significance (Kruskal–Wallis test of one-way ANOVA with Dunn’s multiple comparisons test) and with alpha < 0.05 compared to: negative control *; CpG only positive control **; vehicle-only nanoparticles ^. IP = intraperitoneal.

**Table 2:** Scores of tissue inflammation following intravaginal delivery of curcumin-loaded nanoparticles prior to inoculation with CpG-ODN.

Pre-Treatment	CpG-ODN	Mouse	Pathology Score	Average Score per Mouse (± S.D.)
Water	N	1	0	0.33 (0.58) **^,^^
		2	0	
		3	1	
Water	Y	1	3	2.67 (0.58) *
		2	3	
		3	2	
Vehicle-Only Nanoparticles	Y	1	1	1.67 (0.58) *
		2	2	
		3	2	
Curcumin Nanoparticles	Y	1	0	0.50 (0.58) **^,^^
		2	1	
		3	0	
		4	1	

Statistical significance (Kruskal–Wallis test of one-way ANOVA with Dunn’s multiple comparisons test) and with alpha < 0.05 compared to: negative control *; CpG only positive control **; vehicle-only nanoparticles ^.

**Table 3:** Cumulative pathology for mice pre-treated intravaginally with curcumin nanoparticles and infected with high- or low-dose HSV-2.

Pre-Treatment	HSV-2 Infection Dosage	Number of Mice	Days with No Pathology	Cumulative Pathology Score	Average Score per Mouse
Water	high	4	1–3	75	18.8
Vehicle-only Nanoparticles	high	5	1–2	98	19.6
Curcumin Nanoparticles	high	6	1–3	95	15.8
Vehicle-only Nanoparticles	low	5	4–6	53	10.6
Curcumin Nanoparticles	low	5	4–6	53	10.6

Pre-treatment was 6 h prior to HSV-2 infection. Pathology was observed for 8 days for mice receiving IVAG high dose (10^4^ PFU/mouse) and 10 days for those with low dose (10^3^ PFU/mouse).

**Table 4:** Cumulative pathology for mice pre-treated intravaginally with crude or solid dispersion formulations of curcumin and infected with HSV-2.

Pre-Treatment	Number of Mice	Mice with Pathology Score ≥3 by Day 6	Cumulative Pathology Score	Average Score per Mouse
PBS	5	5	182	36.4
Crude Curcumin	5	3	112	22.2
Curcumin in PVP-K30	5	0	60	12.0
Curcumin in Soluplus^®^	5	0	59	11.8

Pre-treatment was 6 h prior to HSV-2 infection. Pathology was monitored for 12 days in all curcumin treated groups and 8 days in PBS control group.

## Abbreviations

HSV-2	Herpes Simplex Virus—Type 2
FGT	Female Genital Tract
HIV	Human Immunodeficiency Virus
TNF-α	Tumor Necrosis Factor Alpha
NFκB	Nuclear Factor Kappa-B
MCP-1	Monocyte Chemoattractant Protein-1
IL-6	Interleukin 6
PLGA	Poly(Lactic-Co-Glycolic Acid)
ODN	Oligodeoxynucleotides
IVAG	Intravaginal
IP	Intraperitoneal
H&E	Hematoxylin and Eosin
WT	Wild Type
PFU	Plaque-Forming Unit
PVP-K30	Polyvinylpyroline K30
